# Zileuton Beyond Asthma: A Multifaceted 5-Lipoxygenase Inhibitor With Emerging Therapeutic Potential Across Inflammation, Cancer, Neuroprotection, and Fibrosis

**DOI:** 10.7759/cureus.116354

**Published:** 2026-09-16

**Authors:** Victor D Ivancev, Joshua Iskander, Julia Hanna, Kiran Ghotra, Clark Iorio, Diana V Ivancev

**Affiliations:** 1 School of Medicine, Lake Erie College of Osteopathic Medicine, Erie, USA; 2 Pediatrics, East Carolina University, Greenville, USA

**Keywords:** 5-lipoxygenase, alox5, asthma, colorectal cancer (crc), ferroptosis, leukotrienes, zileuton

## Abstract

Zileuton is a selective, orally active inhibitor of the enzyme 5-lipoxygenase (5-LOX), originally developed and clinically approved for the prophylaxis and chronic management of asthma. Discovered in the 1980s, its unique pharmacological mechanism distinguishes it from other widely used leukotriene receptor antagonists. Rather than blocking downstream receptors, zileuton acts upstream to suppress the overall synthesis of all 5-LOX-derived eicosanoids. It achieves this by directly inhibiting the conversion of arachidonic acid into leukotriene A4 (LTA_4_), thereby halting the subsequent production of both the potent chemoattractant leukotriene B4 (LTB_4_) and the cysteinyl leukotrienes responsible for bronchoconstriction and airway inflammation.

Although its formally approved therapeutic identity has remained largely confined to asthmatic populations, emerging pathophysiological insights demonstrate that 5-LOX-derived lipid mediators participate in a substantially broader array of fundamental biological processes. Beyond mediating reactive airway disease, these leukotrienes and related lipid signaling molecules are critical drivers of leukocyte recruitment, vascular permeability, and systemic oxidative stress. Furthermore, 5-LOX activity has been increasingly implicated in complex cellular dynamics, including tumor-microenvironment interactions, tissue fibrosis, and the cascades of lipid peroxidation. These diverse physiological roles have prompted widespread translational investigation of zileuton in a variety of acute and chronic diseases that are substantially removed from its original clinical indication within the scope of pulmonology.

To date, clinical studies have evaluated the therapeutic efficacy of 5-LOX inhibition in several inflammatory conditions, including chronic obstructive pulmonary disease (COPD), ulcerative colitis, rheumatoid arthritis, Sjögren-Larsson syndrome, atopic dermatitis, and acne, as well as in the chemoprevention of pulmonary carcinogenesis. Concurrently, a robust and expanding preclinical literature suggests significant potential for zileuton activity across an even wider spectrum of pathologies. This includes promising experimental efficacy against colorectal malignancy. Additionally, models have highlighted its neuroprotective potential in ischemic and traumatic neurologic injury and ferroptosis-associated neuronal damage. Systemic organ protection has also been observed in models of hepatic fibrosis, ischemic kidney injury, and drug-associated nephrotoxicity. By synthesizing these diverse findings, this review examines the underlying pharmacologic basis and the emerging translational evidence supporting the therapeutic repurposing of zileuton well beyond its traditional role in asthma.

## Introduction and background

Zileuton is a direct inhibitor of 5-lipoxygenase (5-LOX), encoded by ALOX5, and is approved in the United States for the prophylaxis and chronic treatment of asthma in adults and children aged 12 years and older [1]. Its conventional therapeutic identity is therefore unusually narrow. The drug is neither an acute bronchodilator nor a general anti-inflammatory medication. Instead, its established use derives from inhibition of leukotriene biosynthesis, thereby reducing lipid mediators that participate in bronchoconstriction and airway inflammation [2].

Arachidonic acid released from membrane phospholipids can enter several enzymatic pathways. Within leukocytes, 5-LOX interacts with 5-LOX-activating protein and converts arachidonic acid to 5-hydroperoxyeicosatetraenoic acid and subsequently leukotriene A4 (LTA_4_) [3]. LTA_4_ acts as a branch point, from which LTA_4_ hydrolase produces leukotriene B4 (LTB_4_), a chemoattractant and activator of neutrophils and other leukocytes. LTC_4_ synthase produces LTC_4_, which is sequentially metabolized to LTD_4_ and leukotriene E4 (LTE_4_). These cysteinyl leukotrienes promote vascular permeability, airway smooth-muscle constriction, mucus production, and inflammatory signaling [4, 5]. Figure 1 displays the mechanism of action of zileuton.

**Figure 1:**
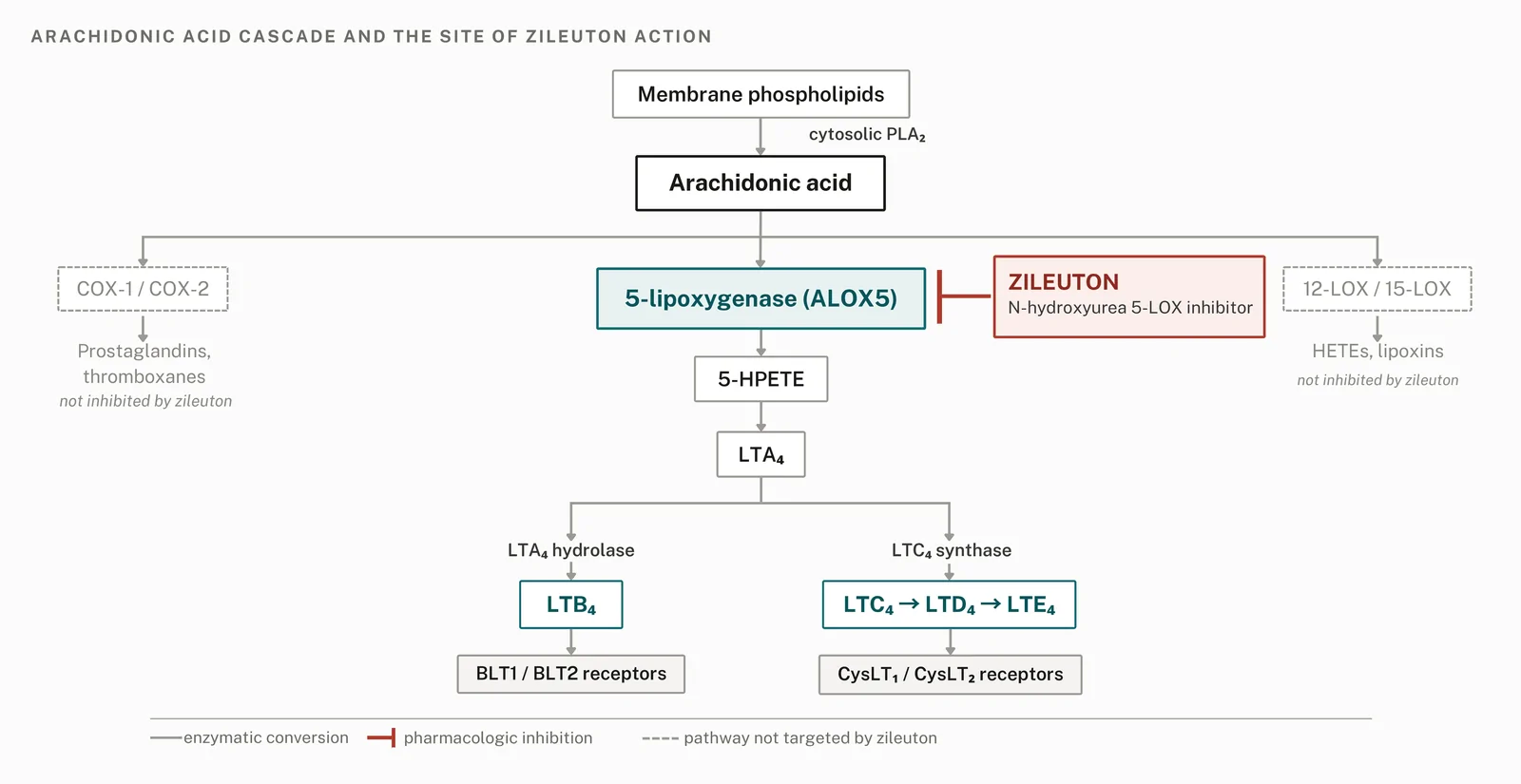
Mechanism of action of zileuton. Cytosolic phospholipase A₂ liberates arachidonic acid from membrane phospholipids. Arachidonic acid is metabolized by cyclooxygenases, by 12-/15-lipoxygenases, or by 5-lipoxygenase (5-LOX, ALOX5), which acts in concert with 5-lipoxygenase-activating protein (FLAP) at the nuclear membrane to form 5-hydroperoxyeicosatetraenoic acid (5-HPETE) and then leukotriene A4 (LTA₄). LTA₄ is converted by LTA₄ hydrolase to leukotriene B4 (LTB₄), a potent neutrophil chemoattractant acting at leukotriene B4 receptor type 1 (BLT1) and leukotriene B4 receptor type 2 (BLT2), or by leukotriene C4 (LTC₄) synthase to the cysteinyl leukotrienes LTC₄, leukotriene D4 (LTD₄), and leukotriene E4 (LTE₄), which act at cysteinyl leukotriene receptor 1 (CysLT₁) and cysteinyl leukotriene receptor 2 (CysLT₂) to produce bronchoconstriction, vascular hyperpermeability, and mucus hypersecretion. Zileuton, an orally active N-hydroxyurea, chelates and reduces the catalytic non-heme iron of 5-LOX, blocking the enzyme itself and thereby suppressing all downstream 5-LOX products, including 5-LOX-mediated peroxidation of polyunsaturated fatty acids that contributes to ferroptosis. Leukotriene-receptor antagonists such as montelukast act only at CysLT₁. Mediators derived from cyclooxygenase (COX-1/COX-2), 12-lipoxygenase (12-LOX), and 15-lipoxygenase (15-LOX) are unaffected.

Zileuton’s influence on this pathway makes it fundamentally different from montelukast and related leukotriene-receptor antagonists. Zileuton inhibits 5-LOX and suppresses the synthesis of multiple 5-LOX products rather than antagonizing a selected leukotriene receptor downstream. This broader effect provides several reasons why 5-LOX inhibition may be relevant to a multifaceted therapeutic approach. First, LTB_4_ is a significant leukocyte-recruitment signal that plays a role in many diseases characterized by excessive neutrophilic or macrophage inflammation. Reduction of LTB_4_ generation could therefore interrupt the amplification of inflammatory pathways. Second, 5-LOX signaling interacts with pathways involved in cell survival, proliferation, migration, and tumor-microenvironment communication. Increased ALOX5 activity has been described in several malignancies, and its inhibition could thus have potential oncologic applications for zileuton. Third, leukotriene signaling participates in tissue injury following ischemia and trauma, where inflammatory cell recruitment can worsen secondary damage. Fourth, 5-LOX is connected to the oxidation of polyunsaturated fatty acids, which is relevant to ferroptosis, an iron-dependent form of regulated cell death in which uncontrolled phospholipid peroxidation contributes to cell death [6]. Finally, unresolved inflammatory signaling can promote fibroblast activation and tissue remodeling, thereby suggesting a potential involvement of 5-LOX inhibition in fibrotic disease.

These mechanistic links have expanded interest in zileuton beyond traditional leukotriene-mediated airway inflammation. Experimental observations suggesting that zileuton can influence ferroptotic injury have further broadened this interest. Zileuton has been evaluated in human studies of ulcerative colitis, rheumatoid arthritis (RA), chronic obstructive pulmonary disease (COPD), dermatologic disease, Sjögren-Larsson syndrome (SLS), and tobacco-related carcinogenesis. In other experimental systems, it has been investigated in various malignancies, cerebral ischemia, spinal cord injury (SCI), traumatic brain injury (TBI), neuroinflammation, depression, liver fibrosis, and kidney injury.

The aim of this review is to evaluate the multifaceted pharmacologic effects and potential clinical applications of zileuton beyond its established role in asthma, emphasizing the strength of evidence, analyzing the limitations, and conveying the safety considerations and areas most appropriate for future investigation.

To address this objective, a narrative literature review was conducted to evaluate the pharmacologic effects, clinical evidence, and emerging therapeutic applications of zileuton and 5-LOX inhibition beyond its established role in asthma. Relevant literature was identified through searches of PubMed/MEDLINE, Scopus, and Google Scholar from database inception through August 2026, using combinations of the terms "zileuton," "5-lipoxygenase," "5-LOX," and "ALOX5" with disease-specific and mechanism-specific terms including "asthma," "chronic obstructive pulmonary disease," "ulcerative colitis," "inflammatory bowel disease," "rheumatoid arthritis," "Sjögren-Larsson syndrome," "atopic dermatitis," "acne," "cancer," "carcinogenesis," "colorectal neoplasia," "fibrosis," "kidney injury," "nephrotoxicity," "spinal cord injury," "traumatic brain injury," "cerebral ischemia," "neuroprotection," "lipid peroxidation," and "ferroptosis." Studies were considered for inclusion if they directly investigated zileuton or 5-LOX inhibition in a clinically or mechanistically relevant setting or provided important background information regarding disease-specific pharmacology, drug safety, or clinical practice. Eligible evidence included randomized and non-randomized clinical trials, prospective and retrospective studies, pilot studies, case reports and case series, pharmacokinetic and pharmacodynamic investigations, animal studies, relevant clinical guidelines, and selected reviews. Priority was given to primary studies directly evaluating zileuton, with human clinical evidence emphasized when available and preclinical evidence used to characterize investigational applications for which human data were absent or limited. Duplicate publications, studies lacking direct relevance to zileuton or the 5-LOX pathway, and sources that did not contribute to assessment of pharmacologic rationale, efficacy, or safety were excluded. Findings were synthesized narratively by therapeutic area, with particular attention to distinguishing established clinical efficacy, human investigational evidence, and preclinical evidence. As a result, given the absence of statistical data, a quantitative meta-analysis was not performed.

## Review

Pulmonology

Asthma

Asthma is a heterogeneous chronic respiratory disease characterized by variable respiratory symptoms, airway hyperresponsiveness, and chronic airway inflammation [7]. Patients may experience episodic wheezing that worsens with activity or environmental triggers, dyspnea, chest tightness, and cough. Airflow obstruction results from overlapping processes, including airway smooth-muscle contraction, mucosal edema, inflammatory-cell infiltration, increased mucus production, and, in persistent disease, structural airway remodeling. Leukotrienes contribute to the pathophysiology as bronchoconstrictors and also promote airway edema and hyperresponsiveness. LTB_4_ specifically enhances the activation and recruitment of inflammatory cells [8]. As a result, the pathophysiological mechanism of leukotrienes in asthma provided the biological rationale for developing zileuton as an asthma therapy.

Early randomized clinical studies provided proof that inhibition of leukotriene synthesis could produce measurable therapeutic effects in humans. In a randomized, double-blind, placebo-controlled study of patients with mild-to-moderate asthma, zileuton improved pulmonary function while simultaneously reducing biochemical markers of leukotriene production [9]. Patients receiving 2.4 g/day demonstrated improvement in FEV1, along with reductions in asthma symptoms and beta-agonist use. These are significant findings, as they link the pharmacologic inhibition of the 5-LOX pathway with a measurable improvement in airway function, which provides support for leukotrienes as therapeutic targets in asthma.

Larger and longer trials subsequently demonstrated that these effects could be sustained. In a 13-week randomized study involving approximately 400 patients with mild-to-moderate asthma, zileuton (600 mg four times daily) significantly improved FEV1, asthma symptoms, rescue-medication use, and quality-of-life measures compared with placebo [10]. Patients receiving zileuton were also less likely to require systemic corticosteroids for asthma exacerbations [10]. A subsequent six-month multicenter study similarly demonstrated improvements in FEV1 and peak expiratory flow, reductions in daytime and nighttime symptoms, decreased beta-agonist use, and fewer patients requiring systemic corticosteroid rescue [11]. Zileuton has also been found to improve pulmonary function and peak respiratory flow in aspirin-exacerbated respiratory disease, which is notable given its relationship with excessive cysteinyl-leukotriene generation [12].

Despite its efficacy in recorded literature, zileuton occupies a relatively limited role in modern asthma management. Today, it serves as a controller medication rather than a first-line acute bronchodilator and is not indicated for reversal of bronchospasm during an asthma attack. The contemporary consensus surrounding asthma treatment is centered primarily on inhaled corticosteroid-containing regimens, while zileuton is generally reserved for selected patients in whom leukotriene-pathway inhibition may provide additional benefit [13]. Its use is further limited by hepatotoxicity and various drug-drug interactions, such as altering the metabolism of theophylline, warfarin, and propranolol, requiring additional caution when these agents are used concurrently [14].

Chronic Obstructive Pulmonary Disease

Acute exacerbations of COPD are associated with increased airway inflammation, often with prominent neutrophilic activity, and leukotriene signaling has been implicated in this inflammatory response [15]. These observations provided a reasonable basis for testing whether pharmacologic suppression of the 5-LOX pathway could improve outcomes during acute exacerbations. However, unlike asthma, where leukotrienes have a well-established functional role in bronchoconstriction and airway inflammation, COPD is driven by a broader and more heterogeneous network of inflammatory and structural abnormalities [16]. Given the more broad involvement of pathological mechanisms in COPD, the targeting of a single mediator may not be completely sufficient to combat an acute exacerbation. This hypothesis was evaluated in a multicenter, randomized, double-blind, placebo-controlled trial of patients hospitalized with acute COPD exacerbations [17]. A total of 119 patients were randomized to receive zileuton (600 mg) four times daily or placebo for 14 days, with treatment initiated shortly after hospitalization. The investigators examined whether adding zileuton to standard management could reduce hospital length of stay and improve other clinical outcomes associated with treatment response and recovery, and thus represented an adequate test of whether interrupting leukotriene production during an active inflammatory exacerbation could produce a clinically meaningful benefit. Findings revealed that urinary LTE_4_ concentrations decreased substantially after treatment, with reductions of approximately 75% at 24 hours and 71% at 72 hours compared with baseline, demonstrating effective suppression of cysteinyl-leukotriene production [17]. Despite the clear biochemical evidence of target engagement, the principal clinical outcomes were not significantly improved. The mean hospital length of stay was similar between the zileuton and placebo groups, and the proportion of patients experiencing treatment failure within 30 days was also not significantly different. Changes in pulmonary function likewise failed to demonstrate a convincing treatment effect. Thus, the trial showed that substantial suppression of a biologically relevant inflammatory mediator did not necessarily alter the short-term clinical course of an acute COPD exacerbation.

Several factors complicate interpretation of these findings. The trial was terminated before reaching its planned enrollment because of slow recruitment, limiting its statistical power to detect modest clinical effects. Moreover, although zileuton markedly suppressed urinary LTB_4_, no significant between-group reduction in serum LTB_4_ was demonstrated at 30 days. This distinction may be relevant given the prominent role of LTB_4_ in neutrophil chemotaxis in COPD, including evidence that LTB_4_ contributes substantially to the chemotactic activity of airway secretions during acute exacerbations [18]. Furthermore, patients hospitalized with COPD exacerbations can differ substantially in the dominant inflammatory mechanisms driving their disease, including bacterial or viral infection, eosinophilic inflammation, neutrophilic inflammation, and non-inflammatory triggers. It therefore remains possible that a subgroup characterized by particularly strong leukotriene-pathway activation could respond differently from an unselected hospitalized population, although this has not been demonstrated clinically.

Gastroenterology

Inflammatory Bowel Disease

Inflammatory bowel disease encompasses ulcerative colitis and Crohn's disease, both of which are characterized by chronic intestinal inflammation, although the direct clinical investigation of zileuton has centered predominantly on ulcerative colitis. Neutrophilic infiltration of the colonic mucosa is a prominent feature of active ulcerative colitis, and early studies identified increased production of LTB_4_ within inflamed intestinal tissue [19]. Human tissue studies further demonstrated greater LTB_4_-dependent neutrophil chemotactic activity in inflamed than in uninflamed ulcerative colitis mucosa [20]. These observations generated early interest in 5-LOX inhibition as a potential therapeutic strategy, particularly because suppression of leukotriene synthesis offered an opportunity to intervene upstream in an inflammatory pathway that appeared to be active directly within diseased intestinal tissue.

The earliest human evidence came from Laursen et al. in 1990, when zileuton, then designated A-64077, was administered to 10 patients with active ulcerative colitis [21]. Investigators measured LTB_4_ and prostaglandin E2 in rectal dialysis fluid before and after a single oral 800-mg dose. Median rectal LTB_4_ concentrations decreased from 4.9 ng/mL before treatment to 1.6 ng/mL after four hours and 0.7 ng/mL after eight hours, before returning toward pretreatment levels by 28 hours. In contrast, prostaglandin E2 concentrations were not significantly altered. Truthfully, this study was not designed to establish clinical efficacy; nonetheless, it provided important evidence that orally administered zileuton could reach the relevant area of interest and selectively suppress mucosal leukotriene generation. In this respect, this study established pharmacologic target engagement outside the respiratory system and strengthened the rationale for determining whether suppression of mucosal LTB_4_ could translate into improvement in active disease.

A subsequent phase II study provided an initial clinical efficacy signal, although the small and uncontrolled nature of the study substantially limited interpretation [22]. Eleven patients with mild-to-moderately active ulcerative colitis received oral zileuton 800 mg daily for 28 days. Clinical assessment incorporated stool characteristics, rectal bleeding, abdominal and rectal pain, urgency, and general well-being, while sigmoidoscopy assessed mucosal vascular pattern, erythema, granularity or ulceration, friability, and exudate. All patients experienced some decrease in disease-related discomfort, and eight of the 11 demonstrated improvement in the gross sigmoidoscopic appearance of the mucosa. However, these clinical and endoscopic findings were not accompanied by histologic improvement, but no significant adverse effects were reported. The investigators therefore regarded the findings as sufficiently encouraging to warrant larger controlled studies, but the absence of a comparator group, very small sample, short duration, and lack of histologic response prevented the study from establishing therapeutic efficacy.

The larger clinical evaluation was considerably less convincing. A six-month, double-blind, multicenter, and multinational trial comparing zileuton 600 mg four times daily with mesalazine 400 mg four times daily and placebo in patients with ulcerative colitis who were already in remission was conducted [23]. At six months, 54% of patients receiving zileuton remained in remission compared with 43% receiving placebo and 63% receiving mesalazine. Although the numerical remission rate with zileuton exceeded that with placebo, the difference was not statistically significant (p = 0.094), whereas mesalazine significantly reduced relapse compared with placebo. Zileuton also did not differ significantly from mesalazine. Thus, the authors concluded that zileuton was not significantly better than placebo for maintaining ulcerative-colitis remission and appeared to have limited therapeutic potential in this setting. These results were particularly important because they demonstrated that the strong biochemical rationale and encouraging findings from small early studies did not translate into sufficiently robust efficacy in a larger controlled clinical evaluation.

Hepatic Fibrosis

Hepatic fibrosis develops in response to chronic liver injury and is characterized by progressive accumulation of extracellular matrix, with activation of hepatic stellate cells (HSCs) representing a central event in the fibrogenic response [24]. Studies have demonstrated that pharmacologic inhibition or genetic disruption of 5-LOX could attenuate experimental liver inflammation and fibrosis, providing the basis for more direct investigation of its role in HSCs [25]. In one study, activated HSCs were found to secrete greater amounts of LTB_4_ and LTC_4_ than quiescent cells, and both leukotrienes promoted HSC activation through ERK signaling [26]. Expression of 5-LOX itself increased during HSC activation, while genetic deletion of 5-LOX reduced the expression of fibrogenic genes. Importantly, the investigators also examined human liver specimens and observed increased 5-LOX expression in tissue from patients with non-alcoholic steatohepatitis and liver fibrosis, with expression largely colocalizing with α-smooth-muscle-actin-positive HSCs [26]. These findings connected 5-LOX signaling not only with experimental hepatic inflammation but also, more specifically, with the activated stellate-cell phenotype responsible for extracellular-matrix deposition.

The same study tested zileuton using a targeted drug-delivery approach. Zileuton was incorporated into cyclic RGD-modified liposomes designed to preferentially target activated HSCs [26]. In fibrotic mice, concentrations of zileuton delivered by this system were approximately 29-fold higher in HSCs than in hepatocytes, and treatment suppressed LTB_4_ secretion as well as the expression of fibrotic markers, including α-SMA, Col1a1, Col3a1, Col5a1, TIMP-1, and PAI-1 [26]. Targeted zileuton treatment reduced collagen deposition, hepatic hydroxyproline content, and fibrosis scores in the carbon tetrachloride model, with similar antifibrotic effects observed in mice with methionine-choline-deficient-diet-induced steatohepatitis. A separate rat study also demonstrated activity of conventional zileuton in high-fat-diet-induced non-alcoholic fatty liver disease, in which treatment inhibited the arachidonic-acid/5-LOX pathway and reduced elevated transaminase levels and histologic NAFLD activity scores [27]. Taken together, these studies support a preclinical effect of zileuton on experimental liver injury and identify activated HSCs as a more specific target for its potential antifibrotic activity.

Nonetheless, intriguingly enough, zileuton itself is associated with a clinically recognized risk of hepatotoxicity. In a 12-month safety study involving 2,458 zileuton-treated patients with asthma, alanine aminotransferase (ALT) elevations of at least three times the upper limit of normal occurred in 4.4% of treated patients compared with 1.0% of patients receiving usual asthma care, with most elevations occurring during the first three months of treatment [28]. This safety profile is particularly relevant to chronic liver disease, where pre-existing hepatic injury may complicate prolonged systemic treatment. The targeted-delivery experiments, however, remain notable because they directly investigated an alternative approach designed to concentrate zileuton within activated HSCs rather than hepatocytes. However, this formulation has been evaluated only in experimental systems and has not established clinical efficacy or safety in patients with hepatic fibrosis. At present, the evidence supporting zileuton for hepatic fibrosis remains preclinical, with the possibility of a potential targeted approach for hepatic fibrosis.

Nephrology

Kidney Injury and Nephroprotection

Kidney injury and fibrosis represent another experimental area in which zileuton has been investigated, particularly in relation to ferroptosis and drug-induced nephrotoxicity. Renal tubular epithelial cells are highly susceptible to oxidative injury and lipid peroxidation, and ferroptosis has increasingly been implicated in both acute tubular injury and progression toward renal fibrosis. This relationship was examined using human renal tubular epithelial HK-2 and HKC-8 cells exposed to the ferroptosis inducer RSL3, as well as a unilateral ureteral obstruction (UUO) mouse model of kidney injury and fibrosis [29]. The combination of zileuton and melatonin reduced RSL3-induced lipid peroxidation and cell death, decreased the expression of the lipid-peroxidation-associated proteins 4-HNE and HO-1, and increased GPX4 expression [29]. In the UUO model, combined treatment also reduced tubular injury, α-SMA and fibronectin expression, and renal fibrosis. These effects were associated with increased protein kinase B (AKT) and mammalian target of rapamycin (mTOR) phosphorylation and NRF2 expression, supporting an interaction between the anti-ferroptotic response and AKT/mTOR/NRF2 signaling.

These findings provide experimental evidence connecting zileuton with inhibition of ferroptosis-associated renal injury, but interpretation requires an important qualification. Zileuton was studied primarily in combination with melatonin, and the investigators reported synergistic effects of the two agents [29]. Although individual treatments were included in portions of the experimental work, the principal improvements in ferroptosis, tubular damage, and fibrosis were attributed to combination therapy. The independent contribution of zileuton to the overall renoprotective effect therefore cannot be inferred to the same extent as it could from a study of zileuton monotherapy. Nevertheless, the study demonstrated activity at relatively low experimental concentrations and placed zileuton within a broader body of research examining inhibition of lipid peroxidation as a potential approach to renal fibrosis [29].

A separate line of investigation has examined zileuton as a nephroprotective adjunct during treatment with nephrotoxic antibiotics. Zileuton was evaluated in rats receiving either amikacin or polymyxin B, two antimicrobial agents whose clinical utility can be restricted by dose-limiting renal toxicity [30]. Animals receiving amikacin or polymyxin B alone developed nephrotoxicity at rates of 90% and 100%, respectively, whereas the addition of zileuton reduced the overall incidence to approximately 30% and significantly delayed the onset of kidney injury at effective doses [30]. Histopathologic examination in the amikacin model supported these biochemical results, as amikacin alone produced substantial tubular cell injury and loss of the brush border, whereas kidneys from animals receiving zileuton concurrently showed greater preservation of tubular architecture. These results provide pilot evidence for zileuton as an adjunct capable of attenuating antibiotic-associated acute kidney injury; however, important limitations include reliance on serum creatinine as the principal biomarker and incomplete characterization of the pharmacokinetic and mechanistic basis for protection.

The nephroprotective mechanism of zileuton was further expanded in another study [31]. Investigators developed parenteral zileuton formulations, characterized serum and renal-tissue pharmacokinetics, and evaluated different dosing strategies against nephrotoxicity induced by vancomycin, amikacin, and polymyxin B [31]. Zileuton distributed into renal tissue, achieved systemic exposures reported to be comparable with those produced by clinically used human doses, and did not accumulate after repeated administration. In the rat models, zileuton reduced antibiotic-associated nephrotoxicity in a dose-dependent manner across all three antibiotics. For example, amikacin-associated nephrotoxicity decreased from 90% without zileuton to 30% with 4 mg/kg once daily and 10% with 2 mg/kg twice daily, while polymyxin-B-associated nephrotoxicity decreased from 100% to 30% with effective zileuton regimens [31]. The study also reported no significant changes in serum creatinine, alanine aminotransferase, or body weight when the parenteral formulations were administered alone at 12 mg/kg daily for 10 days. These findings further support the original pilot study from a probable outcome to experimental formulation and dose-optimization work toward repurposing zileuton as a parenteral adjunct [31].

Rheumatology

Rheumatoid Arthritis

RA is a chronic systemic autoimmune disease characterized by persistent synovial inflammation that can lead to progressive cartilage destruction, bone erosion, joint deformity, and functional impairment [32]. Although lymphocytes and autoantibodies are central to disease pathogenesis, the inflamed synovium also contains macrophages, neutrophils, and other innate immune cells that participate in the amplification of local inflammation [33]. LTB_4_ became an early therapeutic target because of its potent ability to recruit and activate neutrophils. LTB_4_ has been identified in synovial fluid from patients with active RA, and subsequent studies demonstrated significantly higher synovial-fluid concentrations in RA than in osteoarthritis, with LTB_4_ levels correlating with leukocyte numbers and other markers of local immune activity [34]. 

The principal clinical investigation of zileuton in RA consisted of a four-week randomized, double-blind, placebo-controlled trial performed at two academic rheumatology centers [35]. The study was designed not only to assess clinical response but also to determine whether systemic administration of zileuton could substantially inhibit leukotriene production in patients with active inflammatory disease. Pharmacodynamic activity was clearly demonstrated. By the first week of therapy, ionophore-stimulated LTB_4_ synthesis decreased by approximately 70%, from a mean of 191.2 ng/mL to 57.5 ng/mL, and suppression was also observed across the other major products of the 5-LOX pathway. These findings demonstrated that substantial systemic inhibition of leukotriene synthesis was achievable in patients with RA and provided evidence that zileuton engaged its intended pharmacologic target outside the respiratory tract. The clinical findings, however, were less definitive. Measures of disease activity improved among patients receiving zileuton, but improvement was also observed in the placebo-treated group. This prevented a clear distinction between a benefit from treatment and non-specific or placebo-associated changes [35]. Thus, this study revealed evidence of pharmacodynamic activity in the corresponding area of interest, but without substantial clinical improvement.

Oncology

Among the proposed non-asthma applications of zileuton, oncology has generated one of the most diverse bodies of mechanistic research. The 5-LOX pathway has been implicated in several aspects of tumor biology beyond its conventional role in inflammation, including cancer-cell proliferation and survival, resistance to apoptosis, metastatic behavior, and interactions between malignant cells and the surrounding immunologic environment [36]. Increased expression of 5-LOX or production of its lipid mediators has been reported in multiple malignancies, including colorectal and lung cancers, prompting investigation of pharmacologic 5-LOX inhibition as a potential anticancer strategy [37]. Thus, zileuton has been evaluated across various experimental tumor systems to determine its effects on tumor-cell growth, apoptosis, inflammatory signaling, and tumor progression. 

Lung Carcinogenesis and Chemoprevention

Preclinical studies provided early evidence that inhibition of the leukotriene pathway could alter pulmonary carcinogenesis. Zileuton was evaluated together with other leukotriene-pathway inhibitors in female strain A mice exposed to vinyl carbamate, a model in which pulmonary lesions progress from adenoma to carcinoma [38]. Zileuton was administered beginning two weeks after carcinogen exposure, and the higher dietary dose significantly reduced lung-tumor multiplicity at 13 weeks. By 43 weeks, zileuton reduced tumor multiplicity by approximately 28%, while also decreasing tumor size and the yield of carcinomas. The investigators concluded that leukotriene-pathway inhibition not only reduced formation of pulmonary tumors but also slowed progression of adenomas toward carcinoma [38].

The preceding findings subsequently led to a randomized, double-blind, placebo-controlled phase II study in 63 current smokers examining combined low-dose aspirin and extended-release zileuton for 12 weeks [39]. Nasal epithelial samples were collected to assess gene-expression signatures associated with cigarette-smoking exposure, lung cancer, COPD, PI3K signaling, and bronchial squamous dysplasia. The intervention produced minimal modulation of the primary smoking-associated gene-expression signature and similarly did not favorably alter most of the evaluated lung cancer or smoking-related signatures. However, aspirin plus zileuton significantly modulated a bronchial gene-expression signature associated with squamous dysplasia in a favorable manner [39]. Despite these findings, zileuton currently has no established role in lung-cancer prevention, and the available human evidence remains limited to biomarker-based investigation rather than demonstration of reduced cancer incidence or mortality.

Colorectal Neoplasia and Intestinal Polyposis

A study examining 5-LOX expression in human normal colonic mucosa, adenomatous polyps, and colorectal carcinomas found a progressive increase in expression across these tissues [40]. Normal colonic epithelium showed essentially no 5-LOX staining, whereas all 16 adenomatous polyps and all eight colorectal carcinomas examined demonstrated positive expression, with staining becoming more intense and diffuse in malignant tissue. The investigators subsequently evaluated whether pharmacologic inhibition of the pathway could influence colorectal tumor growth. In athymic mice bearing xenografts derived from human LoVo or HT29 colorectal cancer cells, oral zileuton significantly reduced tumor growth compared with vehicle-treated controls, as measured by both tumor volume and final tumor weight [40]. These preclinical findings suggest that inhibition of 5-LOX by zileuton may slow tumor growth in colorectal neoplasia.

A further study evaluated zileuton in the APCΔ468 mouse model of spontaneous intestinal polyposis, allowing the effects of 5-LOX inhibition to be evaluated within an intact tumor-associated inflammatory microenvironment rather than an immunodeficient xenograft system [41]. Mice receiving dietary zileuton for 12 weeks developed approximately 2.6-fold fewer small intestinal polyps and roughly half as many colonic polyps as untreated animals. This reduction in polyp burden was accompanied by decreased serum LTB_4_ and substantial changes in both systemic and polyp-associated inflammation. Zileuton reduced circulating proinflammatory cytokines and inflammatory monocyte populations while also decreasing infiltration of macrophages, myeloid-derived suppressor cells, mast cells, and proinflammatory regulatory T-cell populations within polyps [41]. The investigators additionally identified increased LTB_4_ within human dysplastic colorectal lesions compared with healthy tissue. Thus, the effect of zileuton in this model was associated not only with reduced neoplastic burden but also with extensive modification of the inflammatory environment supporting polyp development [41]. The available evidence therefore supports colorectal neoplasia and intestinal polyposis as preclinical investigational applications of zileuton, with further research needed to determine whether these findings have clinical relevance.

Neurology

SLS

SLS is a rare autosomal recessive neurocutaneous disorder caused by pathogenic variants in ALDH3A2, resulting in deficiency of fatty aldehyde dehydrogenase (FALDH). The disorder classically presents with congenital ichthyosis, spasticity, intellectual disability, and severe pruritus [42]. Unlike many of the inflammatory diseases in which zileuton has been investigated, the rationale for its use in SLS is related to a defined metabolic abnormality. FALDH participates in the degradation of LTB_4_ through its ω-oxidation pathway, and patients with SLS demonstrate impaired LTB_4_ catabolism. Prior research into the role of leukotrienes in SLS found markedly elevated urinary concentrations of LTB_4_ and its metabolite 20-hydroxy-LTB_4_ in patients, whereas these compounds were undetectable in healthy controls [43]. The investigators concluded that LTB_4_ degradation is impaired in SLS and identified FALDH as an important component of its metabolic inactivation. This finding provided a particularly direct rationale for investigating whether reducing LTB_4_ production could alleviate manifestations associated with its abnormal accumulation.

The first clinical report of zileuton in SLS was published in 2000 and involved a single patient treated for five weeks [44]. During treatment, concentrations of LTB_4_ and ω-hydroxy-LTB_4_ decreased, while the severity of pruritus also diminished. The investigators additionally reported favorable behavioral changes and a reduction in the abnormal cerebral white-matter lipid peak detected by proton magnetic resonance spectroscopy, which increased again after treatment was discontinued [44]. Although a single-patient observation could not establish efficacy, the simultaneous biochemical and clinical changes provided preliminary evidence that inhibiting leukotriene synthesis could modify the abnormal leukotriene metabolism characteristic of SLS. Subsequently, a further investigation involved five patients with SLS, who were treated with zileuton for three months and evaluated for biochemical, dermatologic, neurologic, and neuropsychological outcomes [45]. Urinary LTB_4_ and ω-hydroxy-LTB_4_ concentrations decreased during treatment, and pruritus scores also improved significantly (p = 0.006), accompanied by reported improvements in general well-being and background electroencephalographic activity. Neuropsychological testing did not demonstrate significant improvement, although the investigators observed a trend toward faster information processing, and cerebral MRI and proton magnetic resonance spectroscopy findings remained unchanged [45]. This study strengthened the evidence that zileuton extended its effects beyond the alteration of leukotriene metabolism and the reduction of pruritus, but its uncontrolled design and small patient population could not establish firm conclusions regarding clinical efficacy.

These observations were later tested more rigorously in a randomized, double-blind, placebo-controlled crossover trial involving 10 patients with genetically and biochemically confirmed SLS aged 7-40 years [46]. Each patient received zileuton and a placebo during separate eight-week treatment periods separated by a four-week washout interval, allowing each participant to serve as his or her own control. At the group level, zileuton did not produce significant consistent improvements in the principal clinical measures of ichthyosis, pruritus, excoriations, or global disease severity compared with placebo. Substantial variability was observed both within and between patients, and convincing clinical improvement was identified in only one participant. Moreover, there were unexpectedly low baseline urinary LTB_4_ and 20-hydroxy-LTB_4_ measurements that prevented the investigators from confirming a biochemical treatment response in this trial. Hence, this study could not reproduce the positive results from prior investigations. The clinical experience with zileuton in SJS therefore remains limited and mixed, but may retain some evidence in decreasing pruritus and generalized well-being.

SCI and TBI

Traumatic SCI and TBI consist of an immediate mechanical injury followed by a secondary cascade that can continue to damage initially surviving neural tissue [47]. In SCI, secondary injury involves ischemia, vascular disruption, edema, excitotoxicity, oxidative stress, lipid peroxidation, inflammatory-cell infiltration, and apoptotic and other forms of cell death. Similar processes occur after TBI, where blood-brain barrier (BBB) disruption, neuroinflammation, excitotoxicity, metabolic dysfunction, oxidative stress, and progressive neuronal loss contribute to secondary brain injury [48]. These delayed processes have generated interest in pharmacologic approaches capable of limiting inflammatory and oxidative injury after the primary trauma. Experimental evidence implicating arachidonic-acid metabolism and 5-LOX-derived mediators in these secondary responses provided the basis for investigating zileuton in models of neurotrauma.

Early evidence for zileuton in SCI was reported using a mouse model in which spinal cord trauma was produced by vascular-clip compression at the T6-T7 level [49]. SCI produced substantial tissue injury and edema accompanied by neutrophil infiltration, increased tumor necrosis factor-alpha (TNF-α) and cyclooxygenase-2 (COX-2) expression, elevated PGE2 and LTB_4_ production, ERK1/2 activation, and apoptotic cell death. Treatment with zileuton significantly reduced spinal cord inflammation and histologic tissue injury, decreased neutrophil infiltration, and reduced TNF-α, COX-2, PGE2, LTB_4_, and phosphorylated ERK1/2. Measures of apoptosis, including TUNEL staining and pro- and anti-apoptotic signaling proteins, were also favorably altered. Importantly, these biochemical and histologic effects were accompanied by significantly improved hind-limb functional recovery during the 10-day observation period [49].

More recent work has expanded the proposed protective effects of zileuton in SCI beyond conventional inflammatory signaling. An investigation was undertaken to determine whether zileuton could influence neuronal ferroptosis, a form of regulated cell death associated with iron-dependent lipid peroxidation, in both cultured neuronal cells and a mouse model of traumatic SCI [50]. Zileuton reduced ferroptosis and lipid peroxidation in erastin-treated HT22 neuronal cells and similarly reduced markers of ferroptotic injury and inflammation within injured spinal cord tissue. Treated mice demonstrated less neurologic damage and improved motor recovery compared with untreated injured animals. These findings are particularly notable given the established role of lipid peroxidation in the secondary injury cascade following SCI; thus, this study connected zileuton treatment with suppression of a specific lipid-peroxidation-associated form of neuronal death rather than inflammatory signaling alone.

The experimental evidence has since extended to TBI. Research reported in 2025 demonstrated that the arachidonic-acid/5-LOX/leukotriene axis was substantially activated following controlled cortical impact injury in mice [51]. Transcriptomic and biochemical analyses demonstrated increased arachidonic acid and LTB_4_ production after injury, together with upregulation of Pla2g4a and 5-LOX that persisted for several days. Single-cell and immunohistochemical analyses localized increased 5-LOX expression predominantly to activated microglia and monocytes rather than neurons or astrocytes. Zileuton treatment reduced leukotriene release, microglial activation, and expression of multiple inflammatory mediators, including IL-1β, CCL2, CCL7, CCR1, and SPP1 [51]. The investigators also observed reductions in lipid reactive oxygen species, neuronal apoptosis, BBB disruption, and histologic brain damage, which were accompanied by improved performance on neurologic behavioral assessments.

Taken together, the studies on SCI and TBI thus far provide a developing but preclinical body of science. There is significant evidence to suggest that zileuton decreased leukotriene production, inflammatory-cell infiltration, tissue injury, apoptosis, and neuronal lipid peroxidation. Despite this mechanistic consistency across experimental models, these findings have not yet established a therapeutic role for zileuton in human neurotrauma. Further investigation is necessary to establish whether these preclinical data may influence clinical outcomes. Nonetheless, this does depict an intriguing potential for zileuton’s usage in neurological and neurovascular trauma.

Neuroprotection and Cerebral Ischemia

Cerebral ischemia occurs when interruption or substantial reduction of cerebral blood flow deprives brain tissue of oxygen and metabolic substrates, initiating neuronal injury within the ischemic core and a broader secondary injury response in surrounding tissue [52]. In addition to the immediate consequences of energy failure, ischemic brain injury involves oxidative stress, excitotoxicity, inflammatory-cell recruitment, cytokine production, BBB disruption, protease activation, and neuronal cell death. Experimental studies have demonstrated increased expression and activity of 5-LOX following cerebral ischemia, providing a basis for investigating whether modulation of this pathway could reduce secondary brain injury [53]. The relevance of leukotriene signaling is also supported by human observational evidence showing increased circulating LTB_4_ concentrations after ischemic stroke that were associated with poorer clinical outcomes [54]. As a result, zileuton has been investigated across several experimental models of focal and global cerebral ischemia.

One of the earliest direct studies conducted utilized rats subjected to permanent middle cerebral artery occlusion (pMCAO) [55]. Oral zileuton administered after the onset of ischemia at 10 or 50 mg/kg significantly reduced cerebral water content and infarct volume at 24 hours. Treatment also decreased expression of both 5-LOX and matrix metalloproteinase-9 (MMP-9) mRNA, while the higher zileuton dose inhibited the active form of MMP-9. This finding is relevant because MMP-9 contributes to the extracellular-matrix degradation and disruption of the BBB during ischemic injury [56].

A subsequent examination of the inflammatory responses was observed more directly in the same permanent focal cerebral-ischemia model [57]. Zileuton significantly reduced neurologic deficit scores and cerebral infarct volume while decreasing myeloperoxidase activity, a marker associated with neutrophil accumulation, and reducing lipid-peroxidation products within ischemic tissue. Treatment also suppressed NF-κB activation, decreased inducible nitric-oxide synthase expression and activity, and reduced circulating TNF-α and IL-1β [57]. Additional work extended these findings to neuronal apoptosis in rats evaluated 72 hours after MCAO. Again, zileuton was found to reduce infarct volume, neurologic impairment, neuronal injury, and TUNEL-positive apoptotic cells while suppressing caspase-1 and cleaved caspase-3 expression [58]. Thus, across sequential studies, zileuton was associated with effects on inflammatory-cell activity, oxidative injury, inflammatory signaling, and apoptotic pathways.

The cerebral-ischemia literature therefore represents a relatively developed preclinical area of investigation for zileuton. Across several rodent studies, treatment has been associated with reductions in infarct volume, cerebral edema, neurologic deficits, neutrophil-associated inflammatory activity, lipid peroxidation, NF-κB signaling, proinflammatory cytokine production, MMP-9 activity, and neuronal apoptosis. Despite the evidence remaining confined to experimental animal models, human studies demonstrating increased LTB_4_ following ischemic stroke provide an investigational pathway toward zileuton’s potential use in cerebral ischemia.

Dermatology

Atopic Dermatitis

Atopic dermatitis (AD) is a chronic relapsing inflammatory skin disease characterized by eczematous lesions, dysfunction of the epidermal barrier, and often severe pruritus [59]. Biologically active concentrations of LTB_4_ have been demonstrated in lesional skin from patients with AD, and experimental studies have shown that LTB_4_ can promote recruitment of inflammatory leukocytes to the skin, providing a rationale for investigating leukotriene-pathway inhibition in this disease [60]. Direct clinical evidence for zileuton began with a pilot study involving six adults with AD who discontinued corticosteroids and antihistamines before receiving zileuton 600 mg four times daily for six weeks [61]. Disease dissatisfaction scores decreased from a mean of 8.0 to 4.4 (p = 0.03), while objective erythema scores decreased from 24 to 14 (p = 0.03). Pruritus also decreased from 7.3 to 4.3, although this did not reach conventional statistical significance (p = 0.06). The study therefore provided an early clinical signal that systemic zileuton could improve both subjective and objective manifestations of AD, but its interpretation is limited by the extremely small sample, absence of a placebo group, short treatment period, and withdrawal of patients’ usual therapies. Subsequent reviews of leukotriene-directed treatment in AD have consequently characterized the evidence for oral zileuton as preliminary rather than sufficient to establish it as a standard therapy.

Later development shifted from systemic zileuton toward topical delivery, an approach intended to target cutaneous inflammation directly while potentially limiting the systemic exposure and hepatotoxicity associated with chronic oral therapy. Q301, a topical zileuton formulation, progressed into phase II clinical development for AD. In a randomized phase IIa trial, Q301 cream was superior to a vehicle for improvement of AD, providing controlled clinical evidence that topical 5-LOX inhibition could produce a dermatologic treatment effect [62]. Thus, zileuton does not currently have an established therapeutic role in AD, but the dermatology literature is notable given the evolution from a small oral study to a specific, topical formation in controlled phase II testing. The available evidence supports topical zileuton as an experimentally investigated application, and further development would require randomized controlled trials demonstrating durable improvements in AD outcomes relative to the effective topical and systemic therapies currently available.

Acne Vulgaris

Acne vulgaris is an inflammatory disorder of the pilosebaceous unit in which increased sebum production, follicular hyperkeratinization, Cutibacterium acnes, and local immune activation interact in lesion formation [63]. It has been demonstrated that 5-LOX and LTA_4_ hydrolase are expressed by human sebocytes, and increased 5-LOX expression has been demonstrated in acne-involved facial skin [64]. Arachidonic acid stimulates LTB_4_ generation, IL-6 release, and accumulation of neutral lipids in cultured SZ95 sebocytes, whereas zileuton partially inhibits these responses and, with longer exposure, reduces both sebaceous lipid content and IL-6 release [65]. In a pilot study of 10 patients with inflammatory papulopustular acne treated with oral zileuton 600 mg four times daily for three months, the acne severity index declined to 41% of its baseline value by week 12, inflammatory-lesion counts decreased by 29%, and total sebum lipids decreased by 35% [66]. Proinflammatory free fatty acids and lipid peroxides in sebum also decreased, and the magnitude of clinical improvement correlated strongly with reductions in total sebum lipids and free fatty acids [66]. A subsequent single-patient study examining seborrhea provided additional evidence that zileuton could directly suppress facial sebum synthesis, with sebum production returning toward baseline after treatment was discontinued [66]. These findings suggest that zileuton’s experimental anti-acne activity decreased inflammatory signaling and the production of sebum.

A later review reported results from a U.S. phase II multicenter study involving 101 patients with mild-to-moderate inflammatory facial acne [67]. In the subgroup with moderate acne, patients receiving zileuton experienced a mean 41.6% reduction in inflammatory lesions compared with 26.2% with placebo (p = 0.025) [67]. Despite these findings, zileuton did not become an established acne treatment. Current American Academy of Dermatology guidelines recommend therapies including benzoyl peroxide, topical retinoids, antibiotics, hormonal treatments, and oral isotretinoin but do not include zileuton among recommended acne therapies [68]. In the realm of dermatology, zileuton remains a therapy hanging in the balance, with an unusual progression from mechanistic studies to small human trials and a multicenter phase II program, with evidence of both anti-inflammatory and sebosuppressive activity.

Overview

In Table 1, we display the uses of zileuton, both in clinical practice and investigatory procedures, the rationale for its use, representative evidence, the current interpretation of the evidence, and its approval by the Federal Drug Administration (FDA) for each respective disease.

**Table 1:** All approved and investigational uses of zileuton. This table summarizes the investigational status, evidence, and approval of zileuton for a variety of medical conditions. It neither includes every single application or disease zileuton has been investigated for nor should it be utilized for patient care. FDA: Food and Drug Administration; FEV1: forced expiratory volume in 1 second; ICS: inhaled corticosteroid; LTB_4_: leukotriene B4; LTE_4_: leukotriene E4; 5-LOX: 5-lipoxygenase; HSC: hepatic stellate cell; UUO: unilateral ureteral obstruction; FALDH: fatty aldehyde dehydrogenase; AA: arachidonic acid; TBI: traumatic brain injury; MMP: matrix metalloproteinase; BBB: blood-brain barrier; MCAO: middle cerebral artery occlusion; AD: atopic dermatitis; IL-6: interleukin-6; SLS: Sjögren-Larsson syndrome.

Disease	Rationale	Evidence	Interpretation	FDA-Approved
Pulmonology				
Asthma	Leukotrienes contribute to bronchoconstriction, airway edema, mucus production, airway hyperresponsiveness, and inflammatory-cell recruitment [4, 5].	Multiple randomized trials demonstrated improved FEV1, symptoms, rescue-medication use, quality of life, and exacerbation-related outcomes [9-12].	Established clinical indication.Use is limited relative to modern ICS-based regimens by hepatic monitoring and drug interactions [9-12].	✔ Yes
COPD	Neutrophilic inflammation and leukotriene production, particularly LTB_4_, are increased during exacerbations [15].	In 119 hospitalized patients, zileuton markedly reduced urinary LTE_4_ but did not significantly improve hospital stay, treatment failure, or pulmonary function [17].	Human investigation inconclusive. Target engagement did not translate into meaningful clinical benefit [17].	✘ No
Gastroenterology				
Inflammatory Bowel Disease	Increased mucosal LTB_4_ and LTB_4_-dependent neutrophil recruitment suggest leukotriene involvement in intestinal inflammation [19, 20].	Zileuton suppressed rectal LTB_4_; a small uncontrolled study suggested clinical/endoscopic improvement, but a larger maintenance trial was not significantly superior to placebo [21-23].	Human evidence mixed. Biologic activity is established, but durable efficacy is not [21-23].	✘ No
Hepatic Fibrosis & Liver Injury	5-LOX signaling contributes to hepatic stellate-cell activation and fibrogenic signaling [25, 26].	HSC-targeted liposomal zileuton reduced leukotrienes, fibrogenic gene expression, collagen deposition, hydroxyproline, and fibrosis in experimental models [26, 27].	Preclinical. Targeted delivery is experimentally investigated; systemic development is complicated by zileuton-associated hepatotoxicity [26, 27].	✘ No
Nephrology				
Kidney Injury and Renal Fibrosis	Ferroptosis and lipid peroxidation contribute to tubular injury and progression of renal fibrosis [29].	Zileuton plus melatonin reduced ferroptosis, tubular injury, and fibrosis in cellular and UUO models [29].	Preclinical. Independent zileuton efficacy is difficult to establish because the strongest study used combination therapy [29].	✘ No
Antibiotic-associated Nephrotoxicity	Adjunctive zileuton may mitigate renal injury caused by nephrotoxic antimicrobials.	Rat studies demonstrated protection against amikacin, polymyxin B, and vancomycin nephrotoxicity; later work evaluated parenteral formulation, dosing, and renal pharmacokinetics [30, 31].	Preclinical. Actively developed preclinical repurposing application, but human efficacy is unestablished [30, 31].	✘ No
Rheumatology				
Rheumatoid Arthritis	LTB_4_ promotes recruitment and activation of neutrophils within rheumatoid synovium [32-34].	A randomized trial demonstrated ~70% suppression of stimulated LTB_4_ synthesis, but clinical improvement was difficult to distinguish from placebo [35].	Insufficient clinical indication. Human pharmacodynamic activity without established anti-rheumatic efficacy [35].	✘ No
Oncology				
Lung Carcinogenesis	Leukotriene signaling may contribute to pulmonary tumor initiation and progression [36, 37].	Zileuton reduced tumor multiplicity and carcinoma development in mice; aspirin + zileuton later favorably altered a squamous-dysplasia-associated airway signature in smokers [38, 39].	Preclinical plus exploratory human biomarker evidence. No evidence that zileuton prevents clinical lung cancer [38, 39].	✘ No
Colorectal Neoplasia and Intestinal Polyposis	5-LOX activity may support neoplastic-cell growth and the inflammatory tumor microenvironment [40].	Increased 5-LOX was demonstrated in human adenomas/carcinomas; zileuton reduced xenograft growth and intestinal polyp burden in mice [40, 41].	Preclinical. Strong preclinical rationale, but no established human preventive or therapeutic efficacy [40, 41].	✘ No
Neurology				
SLS	FALDH deficiency impairs LTB_4_ degradation, directly linking abnormal leukotriene metabolism to the disease [42, 43].	A case report and five-patient study showed reduced leukotriene metabolites and improved pruritus; a later randomized crossover trial did not demonstrate consistent benefit [44-46].	No clinical evidence. Clinically unproven given the small patient population and lack of support from the later randomized trial [44-46].	✘ No
Spinal Cord Injury	Secondary injury involves inflammation, lipid peroxidation, oxidative damage, apoptosis, and ferroptosis [47, 48].	Mouse studies demonstrated reductions in inflammation, tissue damage, apoptosis, lipid peroxidation, and ferroptosis with improved motor recovery [49, 50].	Preclinical. An investigational application. No established human efficacy [49, 50].	✘ No
Traumatic Brain Injury	Post-traumatic AA/5-LOX/leukotriene signaling may contribute to microglial activation, oxidative injury, BBB disruption, and neuronal death [51].	Experimental TBI studies demonstrated reductions in microglial activation, inflammatory mediators, lipid peroxidation, apoptosis, BBB injury, and neurologic impairment [51].	Preclinical. An early preclinical application. No human therapeutic evidence [51].	✘ No
Neuro-protection and Cerebral Ischemia	5-LOX activity may contribute to post-ischemic inflammation, oxidative injury, MMP activation, BBB disruption, and apoptosis [52-54].	Multiple rodent MCAO studies showed reductions in infarct size, edema, neurologic deficits, inflammatory signaling, lipid peroxidation, and apoptosis [55-58].	Preclinical. Relatively developed preclinical evidence, but no established efficacy in human stroke [55-58].	✘ No
Dermatology				
Atopic Dermatitis	Cutaneous LTB_4_ contributes to inflammatory-cell recruitment and skin inflammation [60].	A small oral pilot study showed clinical signals; topical zileuton cream later advanced to controlled phase II testing [61, 62].	Human investigational evidence. Compelling evidence past preclinical data, but not an established AD therapy [61, 62].	✘ No
Acne Vulgaris	5-LOX activity in sebocytes contributes to LTB_4_, IL-6, inflammation, and sebaceous-lipid synthesis [64, 65].	A 10-patient study showed reductions in acne severity and sebum; a later reported phase II study showed greater inflammatory-lesion reduction than placebo in moderate acne [66-68].	Human investigational evidence. Anti-inflammatory and sebo-suppressive activity established but no clinical role was established [66-68].	✘ No

## Conclusions

Zileuton represents an unusual example of an established drug whose pharmacologic relevance extends beyond the indication for which it was originally developed. Although asthma remains its only FDA-approved therapeutic indication, inhibition of 5-LOX affects a signaling network involved not only in bronchoconstriction and airway inflammation but also in leukocyte recruitment, oxidative injury, lipid peroxidation, ferroptosis, tumor biology, and fibrotic remodeling. Human studies in COPD, ulcerative colitis, RA, Sjögren-Larsson syndrome, AD, and acne vulgaris demonstrate that zileuton can exert measurable biologic effects across multiple organ systems, but these studies have generally been limited by small sample sizes, inconsistent efficacy, or a lack of meaningful clinical benefit. This distinction is particularly evident in COPD, ulcerative colitis, and RA, where substantial suppression of leukotriene activity did not establish a durable therapeutic role. In contrast, the preclinical literature continues to show activity in cerebral ischemia, SCI, TBI, hepatic and renal fibrosis, antibiotic-associated nephrotoxicity, and malignancies, suggesting that the consequences of 5-LOX inhibition are highly dependent on the particular disease context.

Despite the extensive preclinical rationale and isolated clinical signals supporting the repurposing of zileuton, the broader therapeutic repurposing of zileuton is constrained by several substantial limitations. Most pressing are its inherent pharmacological and safety challenges, such as the recognized risk of hepatotoxicity, which necessitates routine hepatic monitoring, and multiple drug-drug interactions involving the altered metabolism of medications such as theophylline, warfarin, and propranolol. Furthermore, robust biochemical target engagement has frequently failed to translate into statistically significant clinical efficacy. In COPD, for example, zileuton markedly suppressed urinary LTE_4_ but did not significantly improve hospital length of stay or treatment failure rates, a finding complicated by early trial termination and the heterogeneous mechanisms driving exacerbations. Similarly, larger controlled trials in ulcerative colitis and rheumatoid arthritis failed to demonstrate superiority over placebo, while initial positive findings in rare conditions like Sjögren-Larsson syndrome could not be reproduced in rigorous, randomized crossover trials. Finally, much of the promising evidence in oncology, neurology, and nephrology remains strictly confined to animal models. The interpretation of these experimental data is often complicated by confounding variables, such as the use of combination therapies (e.g., with melatonin) or a reliance on limited biomarkers, while systemic drug development for hepatic fibrosis is directly impeded by the drug's liver toxicity, necessitating experimental targeted-delivery systems that have yet to establish human safety or efficacy.

Future development of zileuton beyond asthma should therefore be guided by more than the observation that 5-LOX activity is elevated in a particular disease. The most convincing applications will require evidence that 5-LOX signaling is sufficiently important to disease progression, that clinically achievable drug exposure produces meaningful target inhibition within the relevant tissue, and that this pharmacologic effect translates into validated clinical outcomes. Emerging approaches such as tissue-directed drug delivery, combination therapy, biomarker-based patient selection, and investigation of ferroptosis-related mechanisms may help define settings in which zileuton has greater therapeutic relevance. At the same time, any repurposing strategy must account for its established adverse effects, particularly hepatotoxicity, drug-drug interactions, and the feasibility of sustained exposure. Overall, zileuton has potential for a drug previously pushed to the sideline, and with evolving repurposing literature, its future value will depend on identifying diseases in which it may become functionally important and therapeutically beneficial.
